# A comparison of wearable fitness devices

**DOI:** 10.1186/s12889-016-3059-0

**Published:** 2016-05-24

**Authors:** Kanitthika Kaewkannate, Soochan Kim

**Affiliations:** Department of Electrical and Electronic Engineering, Hankyong National University, Anseong, Gyonggi-do South Korea

## Abstract

**Background:**

Wearable trackers can help motivate you during workouts and provide information about your daily routine or fitness in combination with your smartphone without requiring potentially disruptive manual calculations or records. This paper summarizes and compares wearable fitness devices, also called “fitness trackers” or “activity trackers.” These devices are becoming increasingly popular in personal healthcare, motivating people to exercise more throughout the day without the need for lifestyle changes. The various choices in the market for wearable devices are also increasing, with customers searching for products that best suit their personal needs. Further, using a wearable device or fitness tracker can help people reach a fitness goal or finish line. Generally, companies display advertising for these kinds of products and depict them as beneficial, user friendly, and accurate. However, there are no objective research results to prove the veracity of their words. This research features subjective and objective experimental results, which reveal that some devices perform better than others.

**Methods:**

The four most popular wristband style wearable devices currently on the market (Withings Pulse, Misfit Shine, Jawbone Up24, and Fitbit Flex) are selected and compared. The accuracy of fitness tracking is one of the key components for fitness tracking, and some devices perform better than others. This research shows subjective and objective experimental results that are used to compare the accuracy of four wearable devices in conjunction with user friendliness and satisfaction of 7 real users. In addition, this research matches the opinions between reviewers on an Internet site and those of subjects when using the device.

**Results:**

Withings Pulse is the most friendly and satisfactory from the users’ viewpoint. It is the most accurate and repeatable for step and distance tracking, which is the most important measurement of fitness tracking, followed by Fitbit Flex, Jawbone Up24, and Misfit Shine. In contrast, Misfit Shine has the highest score for design and hardware, which is also appreciated by users.

**Conclusions:**

From the results of experiments on four wearable devices, it is determined that the most acceptable in terms of price and satisfaction levels is the Withings Pulse, followed by the Fitbit Flex, Jawbone Up24, and Misfit Shine.

## Background

Nowadays people, are very interested in wearable devices as these are the trend in technology for the tracking of daily life activities. The best activity life trackers on the market today are highly evolved cousins of pedometers. They are smarter and more accurate and can do much more than just calculate how far you walk [1].

A wearable device is a new type of technology in the form of small hardware that includes an application with tracking and monitoring fitness metrics such as distance walked or run, calories consumed, and in some devices heart rate and sleep tracking. The term is now used primarily in reference to dedicated electronic monitoring devices that are synced, in many cases wirelessly, to a computer or smartphone for long-term data tracking. There are also smartphones with the independent ability to track [2]. Wearable devices are tiny, state-of-the-art computers that users wear on various parts of their bodies, such as glasses [3], smart watches, wristbands, or bracelets [4] clipped onto the clothing [5].

Wearable technology has become popular; it allows the wearer to access information in real time. Applications can be used in the fields of health, fitness, food, and aging [1]. Further, it is possible to automate the monitoring and recording of daily activities or fitness. It is also possible to integrate them into more easily worn equipment. The wearable device should monitor workouts and display information about the user’s daily routine on its screen or on a smartphone. This is a more comfortable and convenient method for the wearer than the old method, which required one to calculate the distance or running steps manually.

Reviews of wearable trackers appear on many Internet sites. Often, they show different opinions about the reviewed products. However, these opinions are subjective and do not show any research results that provide the accuracy of information on devices or the identity of the subjects in the experiments or the reviewer. Further, there is no objective data like concrete comparison table to show the results of the subjects reviewed. For example, from “Top Ten Reviews” [7], the best wearable device reviewed was the Fitbit, followed by the Jawbone and Withings Pulse. From this site, the scores were tabulated and compared, but no details were provided about where the information originated. Another example of a wearable tracker review site is “"Best Fitness Tracker". From "TechAdvisor"” [8], the best tracker was Jawbone, followed by Misfit, Fitbit, and Germinly. The reviews on this site do not include a physical comparison table, but, as mentioned on the site, are only reviews from a single blogger. Even though this kind of review website has no objective information, it is advantageous for customers who plan to buy this kind of product because it can help them find the most suitable option. This process may be improved to better benefit customers if the reviews for wearable devices had real objective comparison results, which would help customers best fit their needs.

This paper summarizes and compares the satisfaction, user friendliness, and accuracy of currently popular wearable devices (wristband type) that are found in the top ten of best 2015 fitness trackers according to reviews and comparisons [6–8]. Four wearable devices were chosen randomly from the top ten products in the review comparisons. The four selected wearable devices were the Fitbit Flex (Fitbit Inc., San Francisco, California, USA) [9], Withings Pulse (Withings SA, Issy les Moulineaux, France) [5], Misfit Shine (Misfit Inc., Apple Inc., Apple, Mitten Rd., Burlingame, California, USA) [10] and Jawbone Up24 (Jawbone, San Francisco, California, USA) [11]. Subjective and objective research results will reveal the trackers with the best accuracy and user friendliness based on physical information from real users.

All have multifunction capabilities, such as a step counter, caloric tracker, distance counter, and sleep tracker. The functions are similar, but each device differs in calculation algorithm, user interface, and application. This paper reviews 1) the overall specifications of the four devices—for example, hardware, functions, features in the application on the smartphone; 2) a comparison of the user satisfaction scores; 3) users’ opinions in experiments; 4) reviews of the wearable devices by bloggers or reviewers from Internet sites with a comparison based on a physical information and personal observations by real users; and 5) the accuracy and repeatability of activity tracking for each model.

## Methods

### Wearable devices in experiments

**Fig. 1:**
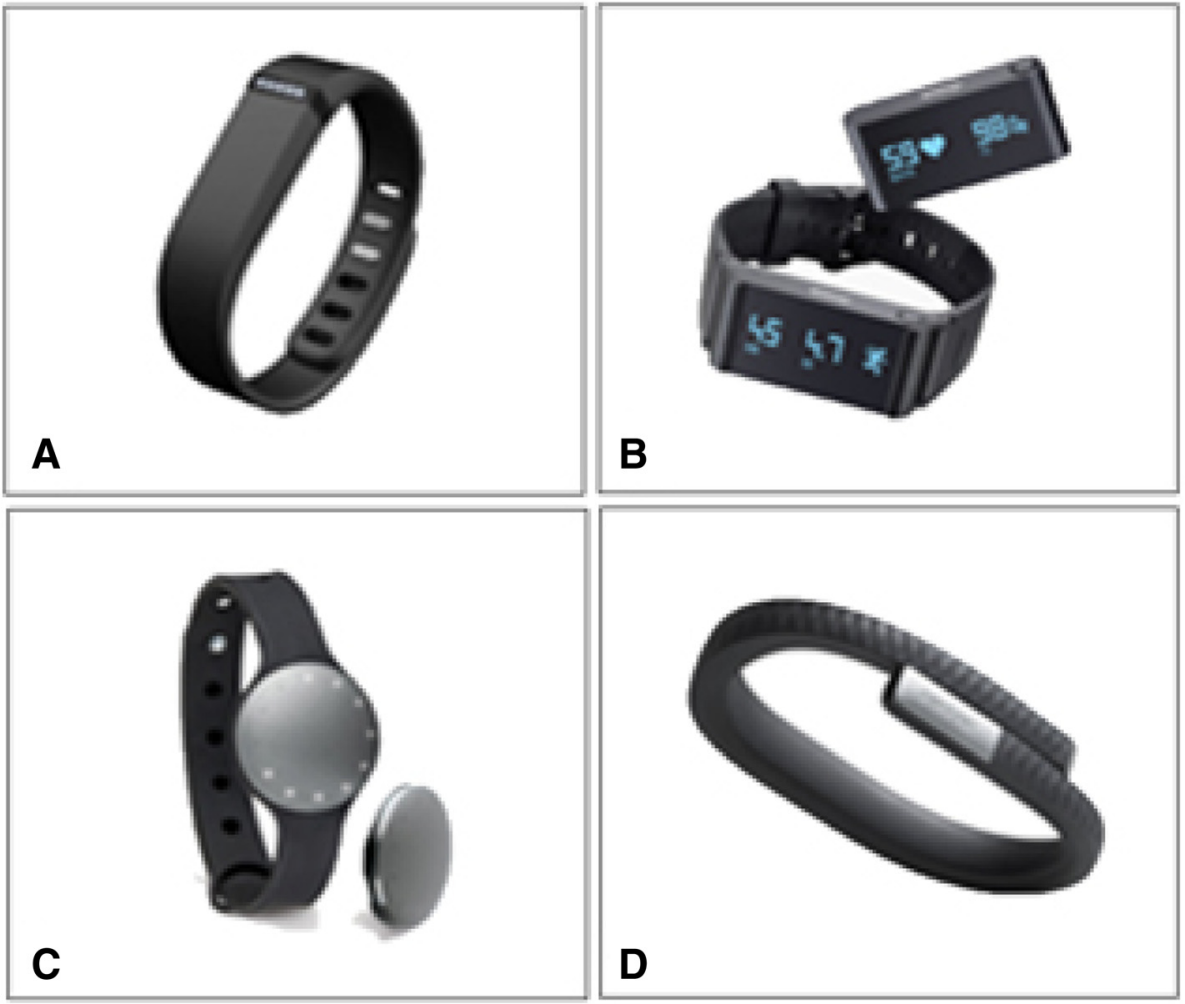
Wearable devices tested. **a** Fitbit Flex, **b** Withing Pulse, **c** Misfit Shine, and **d** Jawbone Up24

The selection of the four wearable devices in the experiments was made randomly for wristband devices available in Korea from among the devices in the top ten review ranking [6, 7] (see Fig. 1). The four devices are described in detail below. Table 1 provides the comparable features of the four wearable devices.

**Table 1:** Comparison of features and functions of four wearable devices

Features	Specifications	Jawbone Up24	Fitbit Flex	Withings Pulse	Misfit Shine
Company Details	Company Name	Jawbone	Fitbit Inc.	Withings SA	Misfit Inc., Apple Inc.
	Country	San Francisco, California, USA	San Francisco, California, USA	Issy les Moulineaux, France	Burlingame, California, USA
	Website	www.jawbone.com	www.fitbit.com	www.withings.com	www.misfit.com
Product Released	Released (US)	13-Nov-13	6-May-13	27-Jun-13	16-Sep-14
	Announced (US)	13-Nov-13	7-Jan-13	6-Jan-13	16-Sep-14
	Present Availability	Available	Available	Available	Available
Type	Smart watch	Watch style	Wearable/clip-on	Wearable/clip-on	Wearable/clip-on
General	Price in Market	$150	$100	$100	$95
	Dimension (W × D × H)	6.1 × 6.1 inch	small: 5.5 × 0.6 inch, large: 6.3 × 8.2 inch	1.7 × 0.87 × 0.33 inch	1.08 × 0.13 × 1.08 inch
	Weight	small: 19 g	small: 16.4 g	8 g	9.4 g
	–	large: 23 g	large: 18.9 g	–	–
Battery	Type	LiMnO_2_ 225 mAh	Lithium polymer battery	Lithium-ion polymer	CR2032 coin cell
	Battery Life	4–6 months	4–6 Months	6 Months	3 Months
	Rechargeable Battery	Yes	Yes	Yes	No
	Changeable Battery	No	No	No	Yes
	Usable Time per Charge (Advertised)	Up to 10 days	Up to 14 days	Up to 14 days	Up to 180 days
	Full Charging Time	3 hours	4 hours	2 hours	No
Tracking Metric (Functions)	Motion	Yes	Yes	Yes	Yes
	Step Counting	Yes	Yes	Yes	Yes
	Distance	No	Yes	Yes	Yes
	Calories	Yes	Yes	Yes	No
	Sleep	Yes	Yes	Yes	Yes
	Heart Rate	No	No	Yes	No
	Fitness Analytics	Yes	Yes	Yes	Yes
	Wind	No	No	No	No
	3D Mapping	No	No	No	No
	Speed	No	No	No	No
	SpO_2_	No	No	No	Yes
	Goal Tracking	No	Yes	No	No
Resistance Function	Water Resistance	Limited	Yes	No	Yes (up to 30 m)
Synchronization	Sync Type	Wireless (Bluetooth)	Wireless (Bluetooth)	Bluetooth	Wireless (Bluetooth)
Connection	Sensor Network	Bluetooth	Bluetooth	Bluetooth	Bluetooth
Screen and Display	Screen Type	Dual LED	5 LEDs	OLED (backlit)	12 LEDs and blink
	Touchscreen	Capacitive finger	Capacitive finger	Capacitive finger	Capacitive touch
	Screen Size (Inch)	No (LED bar)	No (LED bar)	1.69	No (12 LEDs and blink)
Sensor Type	3-Axis Accelerometer	Yes	Yes	Yes	Yes
	3-Gyro Sensor	No	No	No	No
	Magnetometer	No	No	No	No
	Pressure Sensor	No	No	No	No
	GPS	No	No	No	No
	Altimeter	No	No	No	No
Alarm Function		Yes	Yes	Yes	Yes
Data Sharing	–	Yes	Yes	Yes	Yes
Material	Wearable Body Type	Rubber	Rubber	Rubber	Anodized aircraft-grade aluminum
Smart-phone	Smartphone Operating System	iOS 5.1 or greater, Android 4.0 (Ice Cream Sandwich) or later	Windows XP/Vista/7/8	Android 2.3.3 or above, iOS	Pair to iOS only
			Mac OS X 10.5 or above		
			iOS/Android		
UI Interface	History Tracking (Days)	270 days	30 days	10 days	30 days
Social Network Data Sharing	Computer Data Storage (Web App)	No	Yes	Yes	Yes
	Data Sharing	Only friends you already know	Yes	Yes	Yes

1. Fitbit Flex
  The Fitbit Flex (Fitbit Inc., San Francisco, California, USA) is a wristband style, fitness tracking, wireless-enabled, wearable device that measures data including the number of steps walked and the quality of sleep. It can also maintain stimulation and motivation for exercise and movement. The device has several fitness uses and mobile applications. It can be clipped onto a belt or clothing, carried in the pocket, or worn on the wrist as a watch. The market price of the Fitbit Flex is US $100 [9, 10, 12].
2. Withings Pulse
  The Withings Pulse (Withings SA, Issy les Moulineaux, France) is a Wi-Fi-enabled health monitor. The highlight of the Withings Pulse is its ability to measure heart rate and pulse as well as record sleep time displayed as a percentage of the optimal sleep hours. The Withings Pulse can be used for daily life or fitness tracking. The health statistics also include step counting, active calories, and distance travelled. The price of the Withings Pulse is US $120 [5, 6].
3. Misfit Shine
  The Misfit Shine (Misfit Inc., Apple Inc., Burlingame, California, USA) is an activity and sleep tracking monitor that tracks user movements and activity levels. It also tracks daily activities, including step counting, distance travelled, calories burned, and hours of light as well as deep sleep. The Misfit Shine is marketed as a companion to the iPhone and Android app to track fitness goals. The price of the Misfit Shine is US $100 [7, 10].
4. Jawbone
  The Jawbone Up24 (Jawbone, San Francisco, California, USA) is a wearable device that synchronizes wirelessly via Bluetooth, allowing users to track their sleep data, eating habits, calories burned, and daily activity, including step counting and distance travelled. The Jawbone Up24 is designed with only one operating button and has a price of US $100 [7, 11].

### The user interface application (UI app) of each device

Most wearable devices differ in their user interfaces. The UI design for wearable devices should be simple, clear, and quick to navigate for users’ comfort [15]. This can be difficult because wristband type wearable devices are small. Thus, the UI app on a smartphone that links to the wearable device is also an important feature for users. The companion application of a wearable device on a smartphone must be available for easy download. Handheld apps are also useful for heavy processing, analysis, data storage, network actions, or other work [16].

Table 2 shows the comparison of the UI app on smartphones for the four devices explored.

**Table 2:** Comparison of smartphone UI applications of four wearable devices

Features	Jawbone Up24	Fitbit Flex	Withings Pulse	Misfit Shine
Smartphone App Name	Up Tracker	Fitbit	Health Mate (Withings)	Misfit
User Login	Email or Facebook ID	Email	Email	Email or Facebook ID
Make ID and Username	Yes	Yes	Yes	Yes
Goal Setting	Yes	Yes	Yes	Yes
Progress of Activity to Goal	Yes	Yes	Yes	Yes
Daily Report	Yes	Yes	Yes	Yes
Step Counting	Bar graph and number	Bar graph	Bar graph and number	Number
Distance (km)	Number (distance calculated)	Bar graph (number on bar graph)	Number (distance calculated)	Number (distance calculated)
Sleep Tracking	Bar graph and number of average sleep	Sleep log	Sleep bar graph	Sleep log
Sleep Detailed	Sound sleep, light sleep, fell asleep, average bed time, uninterrupted sleep, average wake time	Total sleep, restless, awake	In bed, asleep in, awake, woke up, total sleep	Total sleep, deep sleep, sleep
Weight Progression	Yes	Yes	Yes	Yes
Caloric Analysis	Yes	Yes	Yes	Yes
Food Input	Bar code scan, input type of food	Select food from the list	No	No
Nutrient Analysis in Detail	Yes (show % nutrient info)	No (only calorie calculation)	No	No
Heart Rate Measurement	No	No	Yes	No
Heart Rate Log	No	No	Yes	No
Watch Function	Yes	Yes	Yes	Yes
Fitness Tracking	Show active activity; not specific	Yes (running, weightlifting, others)	No	Yes (swimming and cycling)
Fitness Coach	No	No	Yes	No

### Participants in experiments

Seven healthy subjects participated in the experiments, comprising six healthy men (adults aged 27–50 years, mean age 31 years, mean height 171.5 cm, and mean weight 68.18 kg), and one healthy woman (adult aged 30 years, height 160 cm, and weight 42.1 kg). All participants were graduate students of department of Electrical and Electronic Engineering, Hankyong National University, South Korea. All clinical experiments were carried out from July 2015 to August 2015 with the approval (GIRBA2248) of the Gachon University Institutional Review Board (Incheon, South Korea).

All subjects wore each wearable device for 1 week, changing them after the end of each week. During the use of the devices, all subjects were asked to note the results of use, scores for satisfaction, and opinion about the advantages and disadvantages of each device. The uses of all four devices for one research evaluation were then tested to check and compare the accuracy of each device (the details of the experiments are explained in the following Experimental Methods).

### Experimental methods

1. Satisfaction of subjects using the wearable devices
  In this experiment, each subject wore a wearable device for 1 week, after which they all completed the satisfaction evaluation form, consisting of two sections. (One subject wore the devices for 1 month to test all four devices). The scale satisfaction evaluation form consisted of two sections.
  Section 1. The Likert scale evaluation for each device
    Subjects provided a Likert score for each condition of each device on a maximum five-point scale, according to its general design, features, and functionality after wearing and using it for a week. The scale of satisfaction ranged from one to five points (see Table 3). The two parts of the evaluation form consisted of the following:

**Table 3:** Scale of evaluation and corresponding meanings

Scale (Point)	Meaning
5	very useful and very satisfied
4	moderately useful and moderately satisfied
3	slightly useful and slightly satisfied
2	less useful and less satisfied
1	not useful and not satisfied

**Fig. 2:**
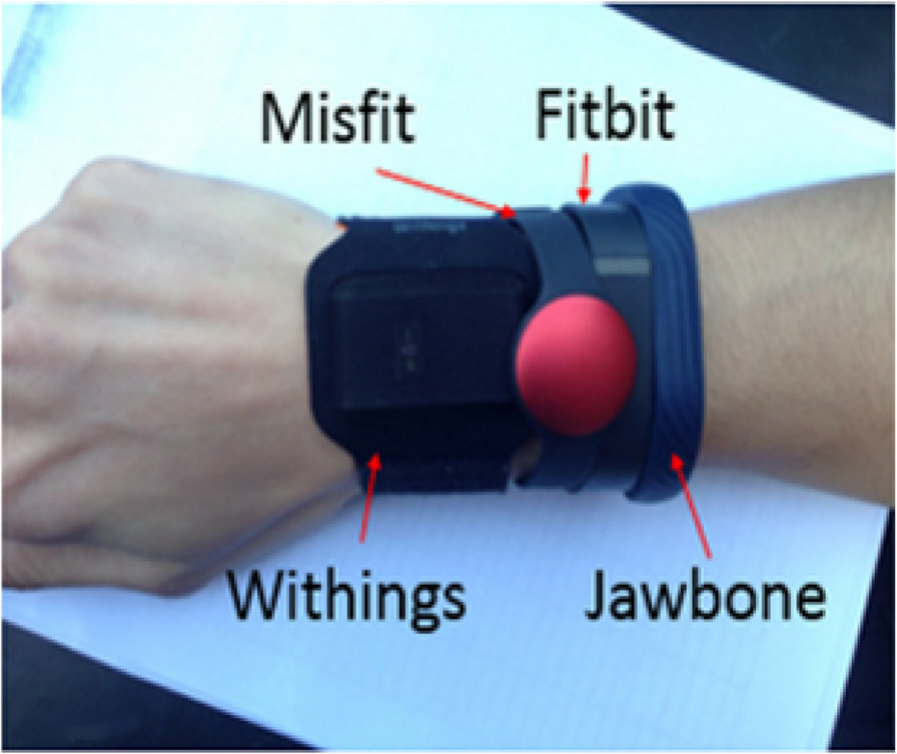
Subjects wore all four devices to measure the accuracy and repeatability of the results
  The repeatability was calculated using Cronbach’s Alpha, SPSS program (SPSS V.2012, IBM Corporation, USA). Subsequently, we scaled scoring among the four devices from best to worst, as defined in Table 4.

      Part 1. The satisfaction score for features and properties
        In this part, the subjects scored their satisfaction with the features and properties of each device. This included the general design (hardware), synchronization, user interface (UI app), battery, friendliness, and ease of use.
      Part 2. The satisfaction score for the metric function of the devices
        In this part, the subjects scored their satisfaction with the metric function of each device. This included step, distance, sleep, and calorie (nutrient) analysis.
  Section 2. Opinion on each device
    In this section, the subjects registered personal comments about the advantages and disadvantages they observed while using each device. Subsequently, the personal opinions and comments from the subjects are shown.
2. Experiment for accuracy and repeatability of each device
  The functions of the wearable devices on the market are similar. However, each device differs in calculation algorithm, user interface, and application. Accuracy and repeatability are two factors that lead the wearer to the real finish line, goal, or diet limit. Nevertheless, other factors include weight, height, age, and gender. Thus, suggesting the best among these four wearable devices requires exploration of the accuracy and repeatability of the devices using objectivemethod and real experimental data.
  The four devices were attached to a subject’s wrist (see Fig. 2). The accuracy and repeatability after testing were measured. The three experiments tested the distance travelled to determine the accuracy and repeatability of all devices.

**Table 4:** Scale of accuracy and repeatability when compared among four devices for each experiment

Scale (Point)	Meaning
4	Highest accuracy or repeatability among the four devices
3	Second highest accuracy or repeatability among the four devices
2	Second lowest accuracy or repeatability among the four devices
1	Lowest accuracy or repeatability among the four devices

    Experiment 1. Distance travelled and step counting of indoor walking
      Subjects wore the devices (Fig. 2) while walking straight across an indoor experiment court. A total distance of 48 m was traversed ten times per person. The data for step counting and distance represented for each device were collected.
    Experiment 2. Distance travelled and step counting of treadmill running (jogging)
      Subjects wore the devices while running or jogging on a treadmill at 8 km/h [13, 14] for 1 min; this was repeated for five trials. The real data record from the treadmill was collected to compare to the real distance calculation from the treadmill’s LCD.
    Experiment 3. Step counting when walking up and down stairs
      Subjects wore the devices while walking up four flights of stairs; this was repeated five times. The subjects then walked down the stairs, which was repeated five times.
      After all data for the experiments were collected, the accuracy and repeatability scores were assigned to the devices on a scale from one to four, with four representing the best accuracy and best repeatability among the four devices (see Table 4).

## Results

### Satisfaction of subjects

After the subjects wore and used each device for a week, they entered the Likert scores into the evaluation form, which included details about the features and properties of the devices, including the UI application. The scale of satisfaction scores is displayed in Table 3.

**Fig. 3:**
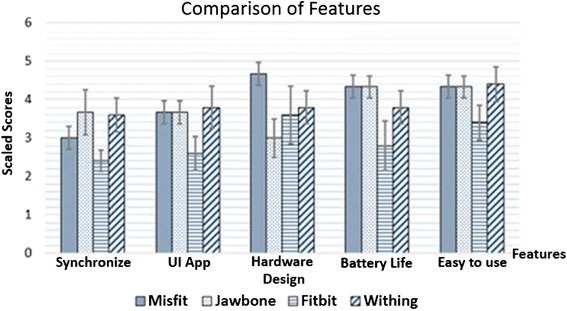
Bar graph comparing mean and standard deviation of the feature satisfaction scores given by subjects when using the devices

**Fig. 4:**
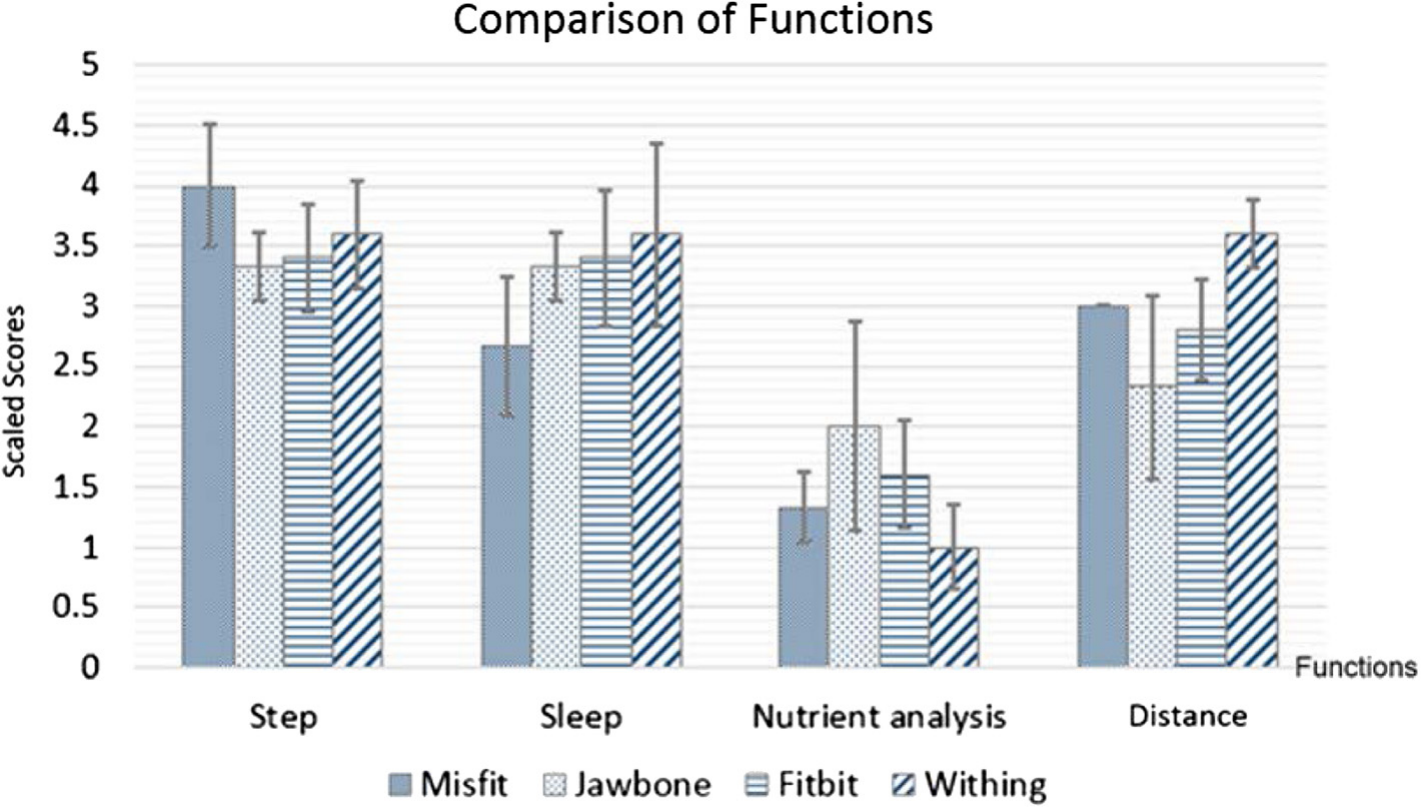
Bar graph comparing mean and standard deviation of the function satisfaction scores given by subjects when using the devices

Figure 3 shows the mean score for the five conditions of features, including device design, battery use, smartphone synchronization, UI applications, and ease of use. Figure 4 shows the mean and standard deviation scores of the satisfaction when using the four main functions of each device, including step counting, sleep tracking, distance tracking, and caloric (or nutrient) analysis. The case of heart rate analysis does not exist in the evaluation score because only the Withings Pulse possessed this function.

From the results, the Withings Pulse had the highest satisfaction score, followed by the Misfit Shine, Jawbone Up24, and Fitbit Flex.

### User feedback

We summarized the opinions of the seven subjects gathered while using the devices. The results in Table 5 show results that came from similar answers from two or more subjects.

**Table 5:** Comparison of opinions (summarized from seven subjects for each device)

Opinion About Features	Jawbone Up24	Fitbit Flex	Withings Pulse	Misfit Shine
Design	Light and good for any sport	Device design is good and sleek, it is good for any sports	Design is not attractive but the fabric band can hold it as a wristband	Design is very attractive, beautiful, and fashionable
Display	Easy to tap the screen to activate	Easy to tap the screen to activate	Display is large and shows the activity tracking without any smartphone sync.	Display is a clock; it can also be used as a watch, but in the sunshine, it is hard to see the LED display
Water Resistance	It is water resistant, but as per the manual, it is less waterproof	It can be used in the shower without worry	As per the manual, it is not water resistant	It is designed for swimming, water resistance is high
UI App	1. Tips on app and how to use always shown on home screen	1. App UI is colorful and fun, easy to use	1. Display is easy to use and colorful	1. Display is beautiful and easy to understand
	2. Enjoyable fitness tracker	2. Nutrient analysis is very detailed	2. Dashboard log is easy to check all activity	2. It has a goal tracker to let you know your daily activity
	3. Dashboard shows the overall daily activity	3. Dashboard shows the overall daily activity	3. The heart rate function is good for checking your health status	3. App can share with your friends and show how your friends are seeking their goals
Metric Function	Sleep tracking is main function and is very detailed but is difficult to use	Food and nutrient calculation is main function and is very easy to use	Pulse O_2_ measurement is main function and can help you detect your heart status	Goal tracking is main function; you can check against your status to seek your goal
Battery	It can charge only through the USB cable	It has a battery indicator to check the battery status, but it has high battery consumption	It has a battery indicator to check the battery status, battery can last many days	It is comfortable, no need to charge the battery
Synchronization	Slow synchronization	Slow synchronization but always loses connection	Fast synchronization, data can be sent via Bluetooth and Wi-Fi	Fast synchronization, but easy to lose connection
Others (Disadvantage/Cons)	1. The device requires smartphone to display	1. It requires smartphone	1. Design is not modern	1. It requires smartphone
	2. No display on itself	2. Slow synchronization	2. If the battery is low, the device cannot connect, data transfer progress on smartphone is inaccurate	2. It has slow synchronization, not always updated in real time
	3. Sleep tracking results are difficult to use and non-automatic	3. The device is confusing, sometimes it needs restarting	3. Sleep tracking is not automatic	3. Sometimes gives inaccurate display
	4. Cannot share data through social networks	4. Tracking problem when climbing or descending stairs	4. Not waterproof	4. Tracking problem when climbing or descending stairs (inaccurate)
	5. Most expensive among four devices	5. High battery consumption	5. Automatic loss of syncing	5. The display does not always respond to finger tapping
	6. It is not fully waterproof	6. Data does not update sometimes	6. Screen is difficult to see in sunlight	6. No nutrient analysis
	7. Slow synchronization	7. Calorie count is not easy to use and only European food included in database	7. No nutrient analysis	7. Always disconnected from mobile phone

From Table 5, it is apparent that all four devices received satisfactory and unsatisfactory feedback from the subjects.

### Additional information (opinions of commercial reviewers on internet sites about the advantages and disadvantage of the devices)

This section is a summarized account of the advantages and disadvantages of devices based on the claims of reviewers on related websites. The selection of review sites was based on the first five listed and ranked on the Google search engine [36] when entering a device name followed by the keyword “review,” such as “Jawbone Up24 review.”

From the first five ranked sites on Google search, it is apparent that these reviews are famous based on the number of interested parties who visit the sites about wearable devices. These claims by reviewers might help customers seeking to buy a device make a choice more easily. Although reviews on websites can be advantageous, nobody can be certain whether the claims are influenced by the manufacturer or are genuine reviews from independent sources. An opinion or claim may come from only one subject or only the reviewer who uses a product.

This is explored to determine whether the pros and cons claimed by the reviewers are similar to the customers’ and seven subjects’ opinions in this study. Tables 6, 7, 8, 9 shows the summarized data of advantages (pros) and disadvantages (cons) for each of the four devices from reviewers on the websites.

**Table 6:** Summary of pros and cons from reviewer opinions for the Jawbone Up24

Reviewer Name	Article Name	Review Date	Reference Site	Advantages (Pros)	Disadvantages (Cons)
Weebly	Jawbone Up24 review	8-Nov-14	[17]	1. Wireless syncing	1. Social sharing: can only add friends you already know
				2. Can use in shower	2. No website interface, only phone app
				3. Deep sleep and light sleep data	3. Hair can become stuck in cap button
				4. “Smart Wake” alarms for naps	4. Overcounts arm movement as steps
				5. Usable design	5. No screen
				6. Holds battery charge for up to seven days	–
Jackson chung	Fitbit Flex vs. Jawbone Up : A comparative review	23-May-2013	[18]	1. Trendy and good design	1. Not accurate tracking
				2. Mobile app is outstanding	2. Only for iOS devices
				3. Battery lasts for 10 days	3. Felt awkward, especially when typing.
				4. Short time charging (only couple of hours)	–
				5. Inexpensive	–
					–
					–
Matt Swider	Jawbone Up24 review	24-Mar-14	[19]	1. Wireless syncing added	1. No display for on-demand stats
				2. Stylish and lightweight	2. Does not have a web app
				3. Very soft rubber for comfort	3. Works with only 10 Android phones
				4. iOS and Android compatible	4. 2.5 mm stereo jack for charging
Michael Sawh	Jawbone Up24 review	26-Mar-14	[20]	1. Bluetooth Smart support for real-time syncing	1. No built-in screen
				2. Slim, stylish design	2. Shorter battery than Jawbone UP
				3. Great silent alarm feature	3. App is sluggish at times
				–	4. Not waterproof
Matthew Miller	Jawbone UP24 review	6-Dec-14	[21]	1. Well-designed band that fits comfortably, long battery life	1. No altimeter to measure stairs climbed
				2. Flawless syncing via Bluetooth	2. Limited just to iOS for now
				3. Integrated Microsoft Office software	3. Hangs up on jackets and long-sleeve shirts
				4. Charges quickly	–
				5. Great sounding front-facing stereo speakers	–
				6. Expandable storage capability	–

**Table 7:** Summary of pros and cons from reviewer opinions for the Withings Pulse

Reviewer Name	Article Name	Review Date	Reference Site	Advantages (Pros)	Disadvantages (Cons)
Weebly	Withings Pulse smart activity tracker review	No mention	[22]	1. Captures heart rate information	1. Not shower safe
				2. Captures flights of stairs climbed and elevation climbed	2. Easy to misplace (leave in pockets, etc.)
				3. Checks running stats (duration and distance travelled) in real time	–
				4. Automatic wireless syncing	–
				5. Captures sleep (duration, quality, light versus deep sleep, interruptions)	–
				6. Screen with constant feedback	–
				7. Discreet and versatile wearing options	–
				8. Battery charge lasts up to 14 days	–
				9. App also pulls in data wirelessly	–
				10. Internet site available for Withings devices	–
Scott Stein	withings Pulse O2 review	25-Apr-2014	[23]	1. Excellent Pedometer	1. Can not read your heart rate without the band on
				2. Excellent Application	2. Screen is not always on
				3. Free iOS and Android apps	3. The screen is not easy to read under sunlight
				4. Accurate heart rate monitoring	4. Not water-resistant
				5. Affordably priced	5. Awkward as a watch
					–
					–
					–
					–
DC Rain-maker	Withings Pulse in-depth review	21-Nov-13	[24]	1. Can record resting heart rate quickly and easily	1. The unit is a bit chubbier than some others
				2. Display is clear and easy to understand	2. Does not track heart rate throughout, only on demand
				3. Good battery life	3. Does not automatically go from sleeping mode to wake mode, must switch manually
				4. Good ability to connect to 3^rd^-party platforms/sites	–
Mikey Campbell	Withings Pulse with built-in heart rate monitor review	4-Nov-13	[25]	1. Variety of sensors	1. Lack of meaningful data presentation
				2. Impressive data accuracy	2. Display lag, touchscreen problems
				3. Flexible carry options	3. Wearability limited to belt clip
Julie Strielmeier	Withings Pulse activity tracker review	23-Aug-13	[26]	1. Size	1. Syncing problem
				2. Can see all important info right on the device itself unlike some devices	2. Sleep data is not always accurate and the detailed data could use some beefing up to show more info
				3. Wireless syncing is a real plus	3. It does not work with a standalone computer
				4. The built-in heart rate sensor is super easy to use	–

**Table 8:** Summary of pros and cons from reviewer opinions for the Fitbit Flex

Reviewer Name	Article Name	Review Date	Reference Site	Advantages (Pros)	Disadvantages (Cons)
Weebly	Fitbit Flex review	10-Aug-14	[27]	1. Comfy wristband form factor	1. Does not track flights of stairs (like the Fitbit One)
				2. Shower-safe water resistance	2. Always visible if worn with short sleeves
				3. Very adjustable wristband	3. No screen on device to show detailed information on goal progress
				4. Progress lights tell you how close you are to reaching your daily goal	4. Very hard to attach to wrist and can pop off (while canoeing, for me)
				5. Wireless syncing	5. Have to tap band repeatedly to enter/exit sleep mode or stop the silent alarm
				6. Great integration with existing fitness apps like MyFitnessPal	6. Chopping veggies can trigger sleep mode
				7. Strong social features including adding friends with a Fitbit device or other Fitbit users, a competition	–
Ben Lippert	Fitbit Flex:Pros and Cons of the wearable activity tracker	13-Aug-13	[28]	1. Best fit for running or walking	1. Fitbit does not play well with bicycles
				2. Tracks everything relating to your activities and sleep, except for stair quantity	2. It is good for any activities that involve free swinging the arms
				3. Battery long last seven days on a single charge	3. It is good when tracking sleep sessions, but sometime the Fitbit going into sleep mode from excessive vibration of the wrist.
				4. Water resistance	–
				5. Good alarm function	–
				6. Calories features is smart	–
					–
					–
					–
Articles by: Suzie	Fitbit Flex review	15-Mar	[29]	1. It is easy to wear all the time	1. Can only charge the tracker with the USB cable
				2. Water resistant	2. It takes a lot of work in the beginning to establish your food menu
				3. Uploads status automatically through Bluetooth or dongle.	3. Sometimes has trouble tapping the tracker into sleep mode
				4. Notification alert to let me know when my battery is running low	–
				5. Learning curve to get the most from it, the dashboard is a colorful and fun display of my activity	–
Bethany Gordon	Fitbit Flex review	Only year mentioned (2015)	[30]	1. Excellent interface	1. This device does not have a screen
				2. Excellent app	2. Only view your data from your computer or your phone

**Table 9:** Summary of pros and cons from reviewer opinions for the Misfit Shine

Reviewer Name	Article Name	Review Date	Reference Site	Advantages (Pros)	Disadvantages (Cons)
Weebly	Misfit Shine activity tracker review	8-Nov-14	[31]	1. Waterproof	1. Sleep data are basic
				2. Wireless data transfer (when placed near device)	2. Shine attachment can come unsecured (can pop out of sports band)
				3. Can track swimming and cycling	3. Time-telling feature suggests it could replace a watch, yet it lacks all other watch features including alerts
				4. Elegant aluminum design	4. Limited info on “screen,” does not have a full digit-based display
				5. On-device feedback to let you know how close you are to reaching a goal	5. Tapping-based interface can be frustrating to use
				6. No recharging. Just replace the watch battery when it runs out (~4–6 months)	–
				7. Partnership with Pebble watch allows you to use the Pebble as a Misfit Shine	–
				8. Social features including a leaderboard, profile, and newsfeed	–
Bethany Gordon	Misfit Shine review	Only year mentioned (2015)	[32]	1. The interchangeable design	1. Tapping the screen is the only way to see your progress
				2. Comfortable band makes it extremely easy to use	2. Does not always respond to tapping
				3. Convenient to wear	3. It has to sit on your arm a certain way to display time and daily progress
				4. Water resistant	–
Kristen Buck	Misfit Shine review	Only year mentioned (2015)	[33]	1. About the size of a quarter and undeniably attractive	1. Only works with iOS
				2. Water resistant	2. Does not have an altimeter to count how many flights of stairs you climb
				3. Great activity monitor for swimmers and surfers	3. Not compatible with Android devices
				4. Can wear it in different ways to track different activities more accurately	–
Jill Duffy	Misfit Shine review	10-Dec-13	[34]	1. Best looking activity tracker	1. Limited data analysis
				2. Includes clip and wristband mounts	2. No integration with other services
				3. Functions as a watch	3. No Web app
				4. Fully waterproof for swimmers	4. No syncing between iOS and Android apps
				-	5. Dashboard lacks weight tracking and calorie counting
Mikey Campbell	Misfit Shine activity monitor review	12-Nov-13	[35]	1. Great design	1. Clunky tagging method
				2. Easy to understand graphical readout	2. Light on features
				3. Long battery life	3. LEDs unusable in bright sunlight

In Tables 5 and 6, matching opinions are shown between the seven subjects and those of the reviewers, implying that the Jawbone Up24 has a good design and fits comfortably. The UI app is colorful and easy to understand. The sleep tracker is very smart and also has good alarm functions. However, disadvantages of the device (cons) include the lack of a screen, inadequate waterproofing, and a complex battery charger.

Tables 5 and 7 display matching opinions between the seven subjects and the reviewers, implying that the Withings Pulse has good primary features, such as the heart rate function. The display is large and can show the tracking results. The data log updates itself via wireless synchronization using Bluetooth. However, the Withings design is not impressive. The display is difficult to see and read in sunlight, and the sleep tracking is not automatic.

Tables 5 and 8 list the matching opinions between the seven subjects and the reviewers, who agree that the Fitbit Flex has a sleek, slim, and good design; is fully water resistant; and has strong social features. However, its weak points include its lack of a screen, difficulty in using the food log and calorie tracking on UI app, and a screen tapping on the device that is sometimes confusing.

Finally, Tables 5 and 9 show the matching opinions between the seven subjects and the reviewers, who agree that the Misfit Shine has an attractive, elegant, and fashionable design. It is highly waterproof and especially good for watersports. The goal tracking motivates the user, and the battery requires no recharge but rather an exchange. However, the Misfit Shine works only with iOS. Android compatibility has been announced, but is not yet available. The display to check the tracking status requires a smartphone. Sometimes, data are inaccurate because of lost syncing to the smartphone.

The most obvious problem among the devices was that all of them experienced automatic loss of synchronization, making it difficult or impossible to update data or resulting in an incorrect report. In contrast, all subjects could use the devices easily and required little to no instruction. This means that the devices were user friendly and easy to use.

### Experiment for accuracy and repeatability of each device

**Fig. 5:**
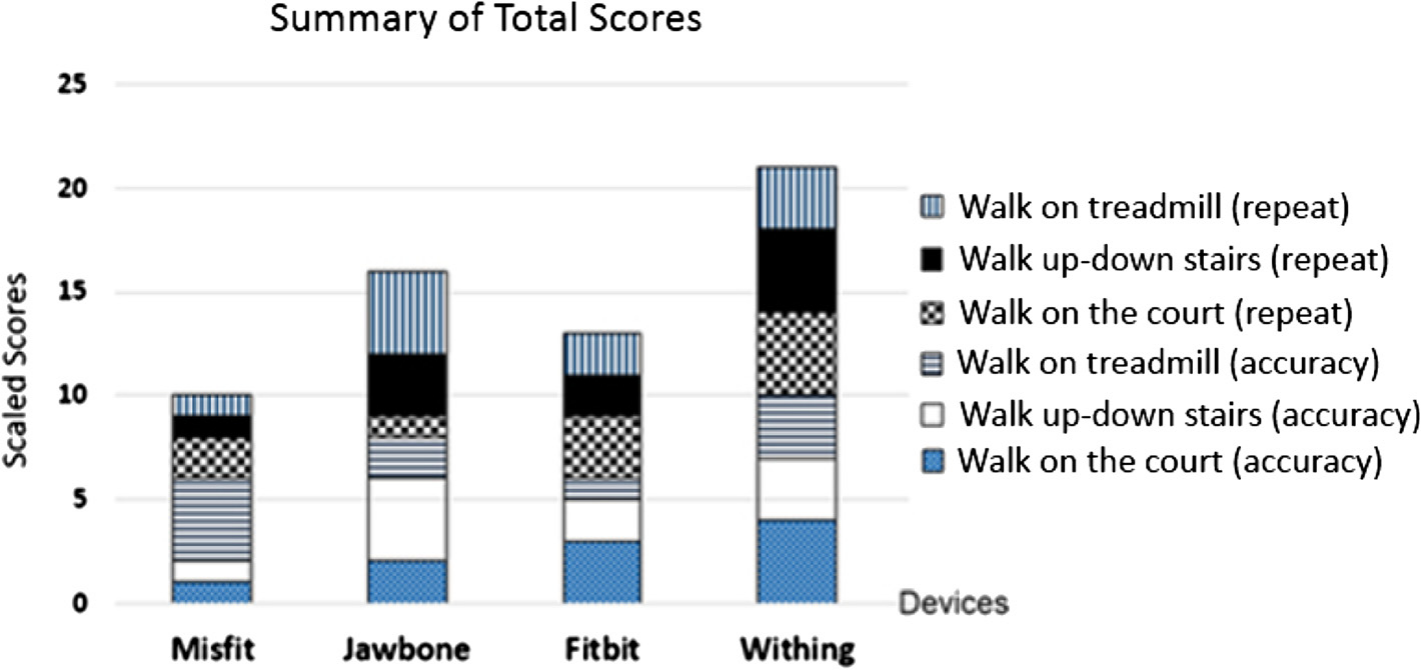
Summary of the accuracy and repeatability scores among the four devices

Table 10 shows that the best device for accuracy and repeatability of indoor walking measurements is the Withings Pulse, with an accuracy of 99.90 % and repeatability of 0.86. The total scores for each device are shown in Fig. 5. The Withings Pulse has the highest score among the four devices for both repeatability and accuracy. The lowest accuracy and repeatability were recorded by the Misfit.

**Table 10:** Comparison of accuracy and repeatability for the devices

Experiments and Results	Devices	Accuracy (%)	Repeatability
Indoor Walking Straight	Jawbone	97.70	0.55
	Withings	99.90	0.86
	Misfit	92.40	0.69
	Fitbit	99.60	0.72
Walking Up/Down Stairs	Jawbone	97.00	0.89
	Withings	97.20	0.83
	Misfit	97.80	0.79
	Fitbit	96.40	0.81
Walking on Treadmill	Jawbone	97.00	0.89
	Withings	97.20	0.83
	Misfit	97.80	0.79
	Fitbit	96.40	0.81

With regard to Table 5 (opinions of the seven subjects) and Tables 6, 7, 8, 9 (opinions of the reviewers), we concluded that the Misfit and Fitbit have difficulty in detecting when a user climbs or descends stairs. In addition to the subject’s experiments in Table 10 and Fig. 5, the scores from experiments with climbing and descending stairs are both lowest in terms of accuracy and repeatability. Thus, the total scores of the Misfit and Fitbit are the lowest among the four tested wearable devices in terms of accuracy and repeatability.

## Discussion

As the results showed, the reason for the low scores earned by the Misfit Shine and Fitbit Flex was stair tracking. These two devices could not track activity when the subjects climbed or descended stairs. For this reason, users were disappointed in these devices.

- Section 1
  - The satisfaction evaluation form considered eight conditions: synchronization, UI app, hardware design, step counting, sleep tracking, nutrient analysis, caloric analysis, battery, and ease of use. The highest satisfaction among the five users of the four devices was gained by Withings, with Misfit, Jawbone, and Fitbit following behind.
  - In addition to Section 1, the opinions of the seven subjects and reviewers on Internet sites were summarized. This showed that each device has different advantages (pros) and disadvantages (cons). However, from the evaluation form and satisfaction scores, the subjective results of real users were shown while using each device. The compared opinions of subjects and reviewers are similar. Thus, we conclude the following:
    - Jawbone Up24 is well designed and fits the subjects comfortably. The UI app is colorful and easy to understand. The sleep tracker is very smart and also has good alarm functions. However, disadvantages (cons) include the lack of a screen, inadequate waterproofing, and a complex battery charger.
    - Withings Pulse has good features such as the heart rate function, which can detect pulse rate. The Withings display is large and can show the tracking results on its display. The data log updates itself via wireless or Bluetooth syncing. However, the Withings design is not impressive: the display is difficult to see and read in sunlight, and the sleep tracking is not automatic.
    - Fitbit Flex has a sleek, slim, and good design; is fully water resistant; and has strong social features. However, its weak points include no screen only Led and a tap screen that is sometimes confusing. The UI app is difficult to use food log and caloric tracking, a steep learning curve.
    - Misfit Shine has an attractive, elegant, and fashionable design. It is fully waterproof and especially good for watersports. The goal tracking function motivates the user to achieve goals, and the battery does not need recharging but rather exchanging. However, the Misfit Shine works only with iOS. Android compatibility has been announced, but is not yet operational. The display to check tracking status requires a smartphone because it has no built-in display. Data are sometimes inaccurate because of lost syncing with the smartphone.
- Section 2
  - The experiments compared the accuracy and repeatability of the four wearable devices. Four points were awarded for the best accuracy and repeatability, and three, two, and one point were given to the second, third, and fourth devices, respectively. The most accurate and repeatable device was the Withings, followed by the Jawbone, Fitbit, and Misfit.
  - In contrast, the Misfit had the highest score for design and hardware. Thus, physical design is also appreciated by users.
  - The Withings was the most friendly and satisfactory from the users’ viewpoint. The Withings was also the most accurate and repeatable for step and distance tracking. The accuracy of tracking is a key measurement for fitness monitoring. The accuracy of personal tracking is different, such as for step count and calories burned, depending on individual measurements such as weight, gender, age, and height. The accuracy of daily tracking activity such as walking, running, or sleeping is important as well. Thus, the objective experiments showed that the Withings was the best device in terms of accuracy and repeatability among the four devices.

## Conclusion

This research attempted to evaluate the best among the four wearable devices selected. This study focused on both objective and subjective methods to obtain results based on physical comparison. The results are independent of manufacturers’ claims. The main two methods of testing verified the quality of the devices, both objectively and subjectively.

From the author’s viewpoint, the most common criticism of wearable devices is that they cannot display information but require a smartphone to send the metric data and reports. The capacity for storage of results is larger in a mobile phone, but it is inconvenient to use both at the same time. Moreover, many fitness tracking applications are presently available through online stores for free without requiring any special or specific hardware. This is very convenient for people who focus on their health or fitness. Although the reports generated by such apps are not guaranteed to be 100 % accurate, they provide the easiest way to track users’ activity without any cost. Thus, the companies that have introduced fitness trackers or wearable devices into this highly competitive market can continually develop new eye-catching products and reduce errors by listening to the feedback and opinions of users from this study to reach a wider market. Technology and aesthetics must go together; unobtrusive designs that are sleek, modern, and lightweight; waterproof functionality; multiple options for recharging the battery; accuracy and repeatability for simple activities such as climbing or descending stairs; and the monitoring of vital parameters (heart rate, pulse rate, body temperature, respiration, or others) should be considered or added. Nonetheless, the present development of wearable devices is moving rapidly with the release of numerous gadgets and new generations. This paper addresses consumer needs with information regarding the performance of four such new gadgets.

### Ethics approval and consent to participate

All clinical experiments were carried out from July 2015 to August 2015 with the approval (GIRBA2248) of the Gachon University Institutional Review Board (Incheon, South Korea). All participant is voluntary. A written informed consent was obtained from each participant. A copy of the signed consent form as well as instructions regarding the fasting period and contact information was delivered to each participant. Also, they have option of withdrawing or discontinuing at any time before and during data collection.

### Availability of data and material

All data sets are available for researcher eligible for access upon request to the corresponding author (sckim@hknu.ac.kr).
